# *In vitro* α-glucosidase inhibitory activity of phenolic constituents from aerial parts of *Polygonum hyrcanicum*

**DOI:** 10.1186/2008-2231-20-37

**Published:** 2012-09-10

**Authors:** Fahimeh Moradi-Afrapoli, Behavar Asghari, Soodabeh Saeidnia, Yusef Ajani, Mobina Mirjani, Maryam Malmir, Reza Dolatabadi Bazaz, Abbas Hadjiakhoondi, Peyman Salehi, Mattias Hamburger, Narguess Yassa

**Affiliations:** 1 Department of Pharmacognosy, Faculty of Pharmacy, Tehran University of Medical Sciences, Tehran, Iran; 2 Department of Pharmacognosy, Faculty of Pharmacy, Mazandaran University of Medical Sciences, Sari, Iran; 3 Department of Phytochemistry, Medicinal Plants and Drugs Research Institute, Shahid Beheshti University, Tehran, Iran; 4 Medicinal Plants Research Centre, Faculty of Pharmacy, Tehran University of Medical Sciences, Tehran, Iran; 5 Institute of Medicinal Plants, ACECR, Tehran, Iran; 6 Department of Medicinal Chemistry, Faculty of Pharmacy, Tehran University of Medical Sciences, Tehran, Iran; 7 Department of Pharmaceutical Sciences, University of Basel, Basel, Switzerland

**Keywords:** *Polygonum hyrcanicum*, Polygonaceae, α-Glucosidase, Antioxidant, Cinnamoylphenethyl amide, Flavonoid

## Abstract

**Background and the purpose of the study:**

The early stage of diabetes mellitus type 2 is associated with postprandial hyperglycemia. Hyperglycemia is believed to increase the production of free radicals and reactive oxygen species, leading to oxidative tissue damage. In an effort of identifying herbal drugs which may become useful in the prevention or mitigation of diabetes, biochemical activities of *Polygonum hyrcanicum* and its constituents were studied.

**Methods:**

Hexane, ethylacetate and methanol extracts of *P. hyrcanicum* were tested for α-glucosidase inhibitory, antioxidant and radical scavenging properties. Active constituents were isolated and identified from the methanolic extract in an activity guided approach.

**Results:**

A methanolic extract from flowering aerial parts of the plant showed notable α-glucosidase inhibitory activity (IC_50_ = 15 μg/ml). Thirteen phenolic compounds involving a cinnamoylphenethyl amide, two flavans, and ten flavonols and flavonol 3-O-glycosides were subsequently isolated from the extract. All constituents showed inhibitory activities while compounds 3, 8 and 11 (IC_50_ = 0.3, 1.0, and 0.6 μM, respectively) were the most potent ones. The methanol extract also showed antioxidant activities in DPPH (IC_50_ = 76 μg/ml) and FRAP assays (1.4 mmol ferrous ion equivalent/g extract). A total phenol content of 130 mg/g of the extract was determined by Folin-Ciocalteu reagent.

**Conclusion:**

This study shows that *P. hyrcanicum* contains phenolic compounds with in vitro activity that can be useful in the context of preventing or mitigating cellular damages linked to diabetic conditions.

## Introduction

The early stage of diabetes mellitus type 2 is associated with postprandial hyperglycemia due to impaired after-meal acute insulin secretion. Hyperglycemia is believed to increase the production of free radicals and reactive oxygen species, leading to oxidative tissue damage and diabetic complications such as nephropathy, neuropathy, retinopathy, and memory impairment [1]. Glucosidases are a group of digestive enzymes which break down the dietary carbohydrates into simple monosaccharides. Glucosidase inhibitors such as acarbose reduce the rate of carbohydrate digestion and delay the carbohydrate absorption from the digestive tract. Therefore, they have a potential to prevent the development of type 2 diabetes mellitus by lowering the after-meal glucose levels [2].

*Polygonum* species are valuable medicinal plants which possess interesting biological activities such as anti-inflammation [3], cardiovascular protection [4], neuroprotection [5], and mitigation of biochemical processes involved in age-related neurodegenerative disorders such as Alzheimer’s [6] and Parkinson’s disease [7]. It is believed that these beneficial effects are, at least in part, due to antioxidant and radical scavenging properties of the plant. Moreover, some *Polygonum* species were reported to possess glucosidase inhibitory properties. Phenylpropanoid glycosides of *P. sachalinense*[8] and tannins of *P. cuspidatum*[9] were subsequently identified as active compounds.

*Polygonum hyrcanicum* is an endemic species that grows widely in northern areas of Iran [10]. In folk medicine of the Turkmen Sahra region (southeast of the Caspian Sea), decoctions made from aerial parts of the plant are used for the treatment of liver problems, anemia, hemorrhoids, and kidney stones [11]. To our knowledge, no biological or phytochemical investigation has been carried out with this species. To explore the plant’s properties with respect to potential prevention or mitigation of cellular damages linked to diabetic conditions, different extracts of *P. hyrcanicum* were tested for α-glucosidase inhibitory, antioxidant and radical scavenging properties. Active constituents were isolated and identified from the methanolic extract.

## Material and methods

### General

Column chromatography was carried out using silica gel (230–400 mesh) obtained from Merck (Germany), RP-18 (230–400 mesh) and Sephadex LH-20 procured from Fluka (Switzerland). Pre-coated silica gel 60 F_254_ plates and silica gel 60 RP-18 F_254_S plates (Merck, Germany) were used for TLC. Spots were observed under UV at 254 and 366 nm and spraying with anisaldehyde-H_2_SO_4_ reagent (Sigma-Aldrich Chemie, Germany) and heating at 120°C for 5 min. HPLC separations were performed on a Knauer Wellchrom system connected to a photodiode array detector (Smart line system, Germany). ^1^H and ^13^C NMR spectra were measured on a Bruker Avance DRX 500 spectrometer operating at 500 MHz for ^1^H and 125 MHz for ^13^C using a 5 mm PABBO probehead. α-glucosidase (EC 3.2.1.20, from baker’s yeast, 77 U/mg), p-nitrophenyl-α-d-glucopyranoside, vitamin E 97% and 2, 2-diphenyl-picrylhydrazyl (DPPH) were obtained from Sigma-Aldrich Chemie (Germany). Sodium carbonate, FeCl3, sodium acetate, ferrous sulfate [FeSO4.7H2O], gallic acid, 2, 4, 6-tripyridyl-s-triazine (TPTZ) solution, and Folin-Ciocalteu reagent were all obtained from Merck (Germany).

### Plant materials

Aerial parts of *Polygonum hyrcanicum* Rech. f. at full flowering stage were collected in September 2008 near the village of Veresk (Mazandaran Province) in the north of Iran. The plant material was identified by the forth co-author. A voucher specimen (6729-TEH) has been deposited at the Herbarium of the Faculty of Pharmacy, Tehran University of Medical Sciences.

### Extraction and isolation

Shade-dried aerial parts of the plant (1200 g) were cut to small pieces and macerated with n-hexane, ethyl acetate, and methanol, successively, at room temperature (3 × 48 hours with each solvent). The extracts were concentrated under reduced pressure, then freeze dried, resulting in dry extracts of hexane (14 g), ethyl acetate (12 g), and methanol (150 g).

Methanol extract (150 g) of *P. hyrcanicum* was suspended in ethyl acetate and divided into an ethyl acetate–soluble portion (ESP, 15 g) and methanol–soluble portion (MSP, 135 g). The ESP was applied to normal phase silica gel column chromatography (5 × 45 cm) and eluted with CHCl3, CHCl3:EtOAc (6:4, 2:8), EtOAc, and MeOH, successively. Seven fractions (ESP_1-7_) were collected. ESP_3_ (375 mg) and ESP_5_ (190 mg) were purified on a Sephadex LH-20 column eluted with MeOH:EtOAc (2:1), to afford compounds **1** (12 mg) and **2** (4 mg), respectively. ESP_6_ (450 mg) was separated on a Sephadex LH-20 column eluted with MeOH:EtOAc (4:1) to give subfractions ESP_6-1_–ESP_6-6_. ESP_6-2_ (42 mg) was subjected to RP chromatography on an RP-18 column eluted with a step gradient of aqueous MeOH (MeOH 40% to 100%). Compounds **3** (11 mg) and **4** (2 mg) were obtained. Compounds **5** (3.5 mg) and **6** (5.5 mg) were purified from ESP_6-3_ (52 mg) by an RP-18 column eluted with aqueous MeOH (50% to 100%). ESP_6-4_ (19 mg) and ESP_6-5_ (28 mg) were separately chromatographed on an RP-18 column eluted with water:MeOH (1:1) to give compounds **7** (5 mg) and **8** (5 mg), respectively.

A portion of the MSP (20 g) was applied to an RP-18 silica gel column eluted with a step gradient of water:MeOH (8:2, 7:3, 5:5, 3:7, 0:10), yielding seven sub-fractions (MSP1–MSP7). Compound 9 (5.6 mg) was obtained by semi-preparative HPLC of MSP3 (128 mg) on an RP-18 column (250 × 20 mm, 7 μm). Water (solvent A) and MeOH (solvent B) were used as mobile phase (0–20 min, 40% B; 20–21 min, 40–50% B; 21-31 min, 50% B; 31–45 min, 50–60% B; 45–46 min, 60–100% B; 46-51 min, 100% B; flow rate of 4 ml/min). Subfraction MSP4 (596 mg) was separated on a Sephadex LH-20 column (MeOH) into four fractions (MSP4-1–MSP4-4). Semi-preparative RP-18 HPLC of MSP4-2 with water (solvent A) and MeOH (solvent B) as the mobile phase (0–30 min, isocratic elution with 50% B; 30–35 min, 100% B, lasting for 5 minutes; flow rate: 4 ml/min) yielded pure **10** (5.4 mg) and **11** (11 mg). MSP_4-4_ was separated on a Sephadex LH-20 column (MeOH) to give compound **12** (7.5 mg). Compound **13** (17 mg) was purified from MSP_5_ (1.148 g) by chromatography on a Sephadex LH-20 column eluted with MeOH–water (8:2) followed by RP-18 chromatography (aqueous MeOH 20–100%). The purified compounds were identified using spectroscopic methods (^1^H and ^13^C NMR, 2D NMR involving COSY, HSQC, and HMBC) and comparison with literature data. The NMR spectra of compound 4 were previously recorded only in acetone-d6 and no ^13^C-NMR data have been reported for compound 5 up to now.

Compound **4**: 1 H NMR (DMSO-d6, 500 MHz); δ = 7.45 (*brs*, H-2'), 7.43 (*d*, *J* = 8.1 Hz, H-6'), 6.92 (*d, J* = 8.1 Hz, H-5'), 6.45 (*brs*, H-8), 6.25 (*brs*, H-6), 5.66 (*brs*, H-1"), 4.74 (br d, J = 4.1 Hz, H-3"), 4.38 (*brs*, H-2"), 4.17 (*dd*, J = 11.7, 3.5 Hz, H-5"), 3.96 (*dd, J* = 11.7, 6.4 Hz, H-5"), 3.81 (*m*, H-4"), 2.13 (*s*, CH3), 1.98 (*s*, CH3); 13 C NMR (DMSO- d6, 125 MHz); δ = 178.0 (C-4), 170.2, 170.5 (COO), 164.5 (C-7), 161.7 (C-5), 157.6 (C-2), 156.7 (C-9), 149.0 (C-4'), 145.5 (C-3'), 133.7 (C-3), 121.5 (C-6'), 121.0 (C-1'), 116.1 (C-2'), 115.6 (C-5'), 108.4 (C-1"), 103.9 (C-10), 99.2 (C-6), 94.0 (C-8), 82.4 (C-4"), 79.4 (C-2"), 79.7 (C-3"), 63.5 (C-5"), 20.7, 20.5 (CH3)

Compound **5**: 1 H NMR (MeOD, 500 MHz); δ = 7.47 (*brs*, H-2'), 7.44 (*d, J* = 8.2 Hz, H-6'), 6.88 (*d*, *J* = 8.2 Hz, H-5'), 6.37 (*brs*, H-8), 6.19 (*brs*, H-6), 5.72 (br s, H-1"), 4.81(*d, J* = 3.8, H-3"), 4.44 (*brs*, H-2"), 3.70 (*d*, *J* = 3.8, H-4"), 3.53 (2 H, H-5"), 2.80 (*s*, CH3); 13 C NMR (MeOD, 125 MHz); δ = 179.4 (C-4), 172.4 (COO), 166.3 (C-7), 163.1 (C-5), 159.6 (C-2), 158.5 (C-9), 149.6 (C-4'), 146.3 (C-3'), 135.0 (C-3), 123.2 (C-1'), 123.0 (C-6'), 117.3 (C-5'), 116.0 (C-2'), 109.8 (C-1"), 105.7 (C-10), 99.9 (C-6), 94.8 (C-8), 86.8 (C-4"), 81.3 (C-3"), 81.2 (C-2"), 62.5 (C-5"), 20.9 (CH_3_).

### Sugar analysis

Sugar moieties of glycoside structures were detected by GC-MS analysis after acid hydrolysis and derivatization with L-cysteine methyl ester and silylation [12].

### α-Glucosidase inhibition assay

α-Glucosidase inhibitory activities were evaluated according to the chromogenic method described by McCue et al. (2005), with some modifications [13]. The enzyme solution contained 20 μl α-glucosidase (0.5 unit/ml) and 120 μl 0.1 M phosphate buffer (pH 6.9). *p*-Nitrophenyl-α-D-glucopyranoside (5 mM) in the same buffer (pH 6.9) was used as a substrate solution. Ten microliters of test samples, dissolved in DMSO at various concentrations, were mixed with enzyme solution in microplate wells and incubated for 15 min at 37°C. Twenty microliters of substrate solution were added and incubated for an additional 15 min. The reaction was terminated by adding 80 μl of 0.2 M sodium carbonate solution. Absorbance of the wells was measured with a microplate reader at 405 nm, while the reaction system without plant extracts was used as control. The system without α-glucosidase was used as blank, and acarbose was used as positive control. Each experiment was conducted in triplicate. The enzyme inhibitory rates of samples were calculated as follows:

$$\begin{array}{ll} \text{Inhibition\%}= & \left[(\text{control absorption}-\text{sample absorption}\right)\\ & /\text{control absorption})]\text{×}100\text{.} \end{array}$$

The IC_50_ values of samples were calculated and reported as the mean ± standard deviation (SD) of three experiments.

### DPPH free radical scavenging activity

The antioxidant activities of the extracts were determined with the DPPH assay according to an established protocol [14]. Extract solutions of 500, 250, 100, and 50 μg/ml were prepared in methanol. Each test tube contained 1 ml of the samples and 2 ml freshly prepared DPPH solution of 40 μg/ml in methanol. Negative control tubes were the same as the test tubes, except that they did not include DPPH. Absorbance of the mixtures were recorded at 517 nm after 30 minutes, against the blank covets of DPPH solution. Vitamin E was used as a positive control. All samples were assayed in triplicate and IC_50_ values were calculated.

### FRAP assay

Antioxidant activities of plant extracts were evaluated by monitoring their ferric-reducing abilities [15]. Freshly prepared FRAP reagent contained 5 ml FeCl3 (20 mM) plus 5 ml of a 10 mM TPTZ solution in 40 mM HCl and 50 ml of 300 mM acetate buffer (pH = 3.6). One hundred microliters of the samples, dissolved in methanol at various concentrations, were mixed with 3 ml FRAP reagent and incubated at 37°C for 10 minutes; the absorptions at 593 nm were recorded. A calibration curve was generated in the range of 125–750 μM ferrous sulfate (FeSO_4_.7H2O). Vitamin E was used as a positive control and the results were expressed as mmol ferrous ion equivalent per gram of extracts.

### Determination of total phenolic content

Total phenolic contents of extracts were assessed using Folin-Ciocalteu reagent [16]. The reagent was diluted 10-fold with distilled water. Two hundred microliters of appropriate dilutions of extracts were added to 1.5 ml reagent and allowed to stand at room temperature for 5 minutes. Sodium bicarbonate solution (1.5 mL, 60 g/L) was added to the mixture and stored at room temperature for an additional 90 minutes; absorptions at 725 nm were recorded. Known concentrations of gallic acid (0–100 μg/ml in methanol) were applied as standard samples and a calibration curve was created. Total phenolic contents were expressed as mg of gallic acid equivalents (GAE) per gram of dry extracts.

## Result and discussion

Sequential extraction of the aerial parts of *P. hyrcanicum* yielded 1.0% of hexane, 0.8% of ethyl acetate, and 12.5% of methanol extracts, respectively. The methanol extract of P. hyrcanicum showed noticeable α-glucosidase inhibitory activity (IC_50_ = 15.3 μg/ml), whereas the ethyl acetate and hexane extracts only caused moderate inhibition. Acarbose (IC_50_ = 8.7 μg/ml) was used as a positive control (Table 1).

**Table 1:** ***In vitro *activities and total phenol content of *P. hyrcanicum *extracts**

**Sample**	**α-Glucosidase inhibition IC**_**50**_**(μg/ml)**	**DPPH assay IC**_**50**_**(μg/ml)**	**FRAP assay (mmol ferrous ion equivalent/g)**	**Total phenol content (mg gallic acid equivalent/g)**
Hexane extract	56.20 ± 1.2	1000<	trace	trace
EtOAc extract	42.30 ± 0.9	146.6 ± 5.2	0.54 ± 0.21	20.30 ± 2.5
MeOH extract	15.30 ± 0.5	76.00 ± 3.4	1.37 ± 0.42	135.00 ± 4.4
Vitamin E	-	14.12 ± 0.9	2.40 ± 0.76	-
Acarbose	8.70 ± 1.1	-	-	-

**Figure 1:**
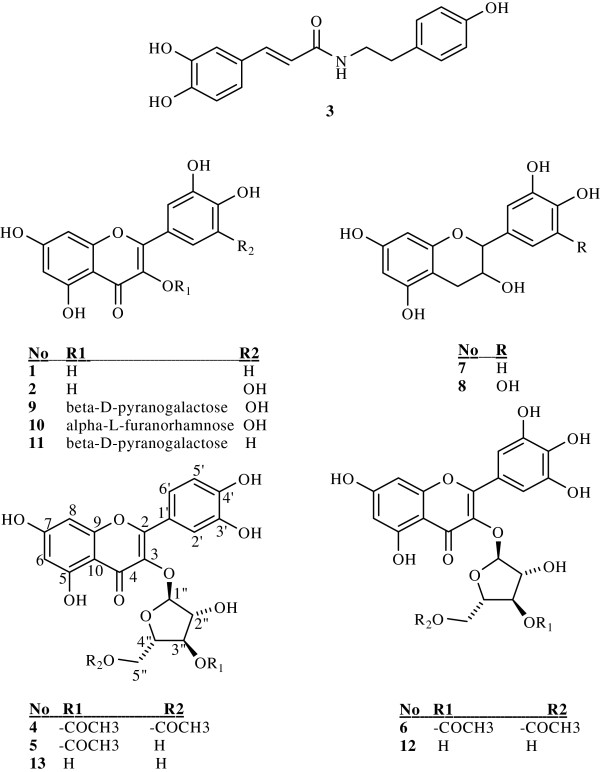
**Structures of the phenolic constituents 1-13 isolated from the methanolic extract of *Polygonum hyrcanicum *Comparisons of the spectroscopic data with literature**[21,26]**led to the identification of sugar moiety of compounds 4, 5, 6, 12, 13.**

In vitro enzyme-inhibitory assay-guided fractionation of methanol extract resulted in the purification of 13 phenolic compounds as the active constituents. Based on NMR data, the purified compounds were identified as quercetin (**1**) [17], myricetin (**2**) [17], N-trans-caffeoyl-tyramine (**3**) [18], quercetin 3-O-α-L-(3",5"-diacetyl-arabinofuranoside) (**4**) [19], quercetin 3-O-α-L-(3"-acetyl-arabinofuranoside) (**5**) [20], myricetin 3-O-α-L-(3",5"-diacetyl-arabinofuranoside) (**6**) [21], (+) catechin (**7**) [22], (-) gallocatechin (**8**) [22], myricetin 3-O-β-D-galactopyranoside (**9**) [23], myricetin 3-O-α-L-rhamnopyranoside (myricitrin) (**10**) [23], quercetin 3-O-β-D-galactopyranoside (**11**) [24], myricetin 3-O-α-L-arabinofuranoside [21] (**12**), and quercetin 3-O-α-L-arabinofuranoside (avicularin) (**13**) [25] (Figure 1). GC analysis of sugars obtained from hydrolysis of compounds 4, 5, 6, 12, and 13 and comparison with the authentic sample resulted in the detection of L-arabinose (RT = 24.3 min). 

The sugar moiety of compounds 9 and 11 were confirmed to be D-galactose (RT = 31.1 min) and compound 10 was verified to have an L-rhamnose (RT = 26.7 min). All the compounds were isolated from P. hyrcanicum for the first time. Compounds 4, 5, and 6 are rare flavonoids with mono- or diacetylglycosyl moieties which have not been detected in the *Polygonum* genus up to now.

Subsequently, α-glucosidase inhibitory activities of phenolic compounds 1–13, isolated from the methanolic extract, were evaluated. The results are reported in Table 2. All constituents showed interesting inhibitory activities while compounds 3, 8 and 10 (IC50 = 0.3, 1.0, and 0.6 μM, respectively) were the most potent ones. The α-glucosidase inhibitory activities of compounds **4**, **5**, **6**, and **12** have not been reported in the literature previously. Comparing the IC_50_ values of tested flavonoids shows that hydroxyl substitution affects the inhibitory activity so that increasing number of free phenolic groups results in higher activity.

**Table 2:** **α-Glucosidase inhibitory activity (IC**_**50**_**) of phenolic compounds 1-13**

**No**	**Compounds**	**IC**_**50**_**(μM)**
1	Quercetin	3.3 ± 2.0
2	Myricetin	1.3 ± 0.6
3	N-trans-Caffeoyl-tyramine	0.3 ± 0.3
4	Quercetin 3-O-α-L-(3″,5″-diacetyl-arabinofuranoside)	4.9 ± 1.5
5	Quercetin 3-O-α-L-(3″-acetylarabinofuranoside)	4.8 ± 1.9
6	Myricetin 3-O-α-L-(3″,5″-diacetyl-arabinofuranoside)	5.8 ± 2.8
7	(+) Catechin	6.6 ± 3.5
8	(-) Gallocatechin	1.0 ± 0.3
9	Myricetin 3-O-β-D-galactopyranoside	4.8 ± 0.8
10	Myricitrin	0.6 ± 0.2
11	Quercetin 3-O-β-D-galactopyranoside	6.7 ± 3.0
12	Myricetin 3-O-α-L-arabinofuranoside	4.2 ± 2.9
13	Avicularin	7.6 ± 0.9
	Acarbose	13.5 ± 1.7

Since oxidative stress is considered as a key factor in the pathogenesis of diabetic complications, antioxidant properties of the extracts were also studied. DPPH radical scavenging activity and ferric reducing power of P. hyrcanicum extracts are summarized in Table 1. The methanolic extract showed noticeable antioxidant activities in both DPPH (IC_50_ = 76.0 μg/ml) and FRAP (1.4 mmol ferrous ion equivalent/g) assays compared to vitamin E (IC_50_ = 14.1 μg/ml, FRAP value of 2.4 mmol ferrous ion equivalent/g) as the positive control. The ethyl acetate extract was a moderate antioxidant, while the hexane extract was not active at the concentrations tested. The antioxidant properties were in accord with the total phenol content of the extracts (Table 1). Methanol and ethyl acetate extracts contained 135.0 mg and 20.3 mg gallic acid equivalent/g, respectively, and the hexane extract was free of phenolic constituents.

## Conclusion

In the perspective of identifying traditional herbal drugs which might be useful in preventing or mitigating cellular damages related to diabetes, we carried out the first study of the Persian edible plant, *P. hyrcanicum*. The methanolic extract, in particular, showed promising α-glucosidase, antioxidant, and radical scavenging activities and thirteen phenolic compounds were purified in an activity-guided approach. All the isolated compounds (IC_50_ = 0.3–7.6 μM) were more potent than the positive control acarbose (IC_50_ = 13.5 μM). This study suggests that P. hyrcanicum is a promising source of active compounds that can prevent the development of diabetes mellitus type 2 and its complications. While these *in vitro* results are of a preliminary nature, further investigation of *P. hyrcanicum*, in particular, *in vivo* pharmacological testing of the methanolic extract is warranted. These studies will provide a more in depth picture on the potential of this interesting traditional Persian plant.

## Competing interests

No conflict of interest has been declared.

## Authors’ contribution

M-AF. Performed plant preparation, extraction, isolation and identification of plant substances and drafted the manuscript. AB. Determined inhibitory activity of the enzyme. SS. Advised separation of plant substances by HPLC. AY. Did the botanical studies and identified scientific name of the Plant. MM. Carried out antioxidant assays and total phenol content of the extracts. MM. Was engaged in phytochemical investigations and helped in isolation of substances. DR. Advised on NMR techniques of isolated compounds. HA. Advised antioxidant assays and edited the article. SP. Advised inhibitory activity determination of the enzyme. HM. Advised sugar analysis and edited the article. YN. Conceived the study and edited the manuscript. All authors read and approved the final manuscript.
